# Supplementary material for: Sex Differences in Serious Adverse Events Reported Following Booster Doses of COVID-19 Vaccination in Thailand: A Countrywide Nested Unmatched Case-Control Study
Source: Vaccines (Basel). 2023 Nov 28;11(12):1772. doi: 10.3390/vaccines11121772 (PMC10747632; doi:10.3390/vaccines11121772)
Supplement: Supplementary file 1 [file vaccines-11-01772-s001.zip › vaccines-2632345-supplementary.pdf]

Table S1 Criteria of serious adverse events based on the AEFI-DDC program (21)

1. Death
2. Life-threatening diseases<br>The following diseases were life-threatening if they were following COVID-19 vaccination:<br>- Anaphylaxis, Stevens-Johnson syndrome<br>- Acute myocarditis, acute pericarditis, acute myocardial infarction, hypertensive emergency, ischemic heart disease, heart failure<br>- Acute respiratory distress syndrome (ARDS)<br>- Transverse myelitis, Guillain Barre Syndrome (GBS), acute disseminated encephalomyelitis (ADEM), Encephalitis, Stroke, Meningoencephalitis, Meningitis, Bell's palsy<br>- Serious immune-mediated diseases such as immune thrombocytopenic purpura (ITP)<br>- Vaccine induced-immune thrombotic thrombocytopenia (VITT), pulmonary embolism, deep vein thrombosis
3. Resulting in permanently handicapped
4. Congenital anomaly<br>*Not included because of unknown pregnancy status
5. Prolong admission for vaccine-related adverse events in the hospital above 3 days<br>*Not included because of unable to confirm causality from the vaccines

Table S2 Definition and example of booster regimens among study population

Booster regimens	Definition	Most common	n (%) (N=2,450)
Heterologous doses	Brand of the booster dose differs from the first difference primary doses	SI-AZ-PZ<br>SI-AZ-MO	559 (22.8%)<br>74 (3.02%)
Heterologous booster dose	Brand of the booster dose differs from the first same primary doses	AZ-AZ-PZ<br>SI-SI-PZ<br>SI-SI-AZ<br>AZ-AZ-MO<br>SI-SI-MO	579 (23.6%)<br>279 (11.3%)<br>226 (9.2%)<br>135 (5.5%)<br>119 (4.7%)
Homologous booster dose	Brand of the booster dose is same as the first same primary doses	SI-AZ-AZ<br>PZ-PZ-PZ	232 (9.5%)<br>110 (4.5%)

AZ:AstraZeneca, PZ:Pfizer, MO:Moderna, SI: Sinovac or Sinopharm

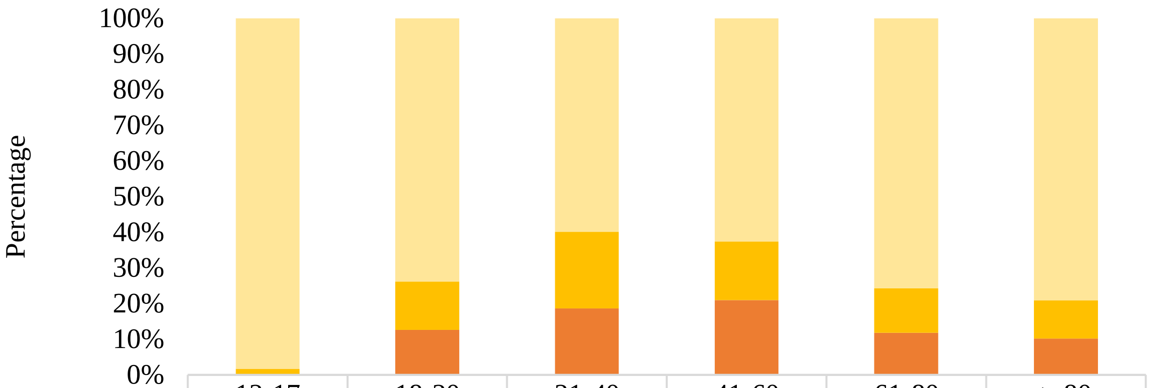

	12-17	18-20	21-40	41-60	61-80	> 80
Pfizer	98.34%	73.80%	59.90%	62.54%	75.74%	79.08%
Moderna	1.54%	13.58%	21.49%	16.46%	12.46%	10.72%
AstraZeneca+	0.12%	12.61%	18.61%	21.00%	11.80%	10.20%

Age group (year)

AstraZeneca Moderna Pfizer

+AstraZeneca was not recommended in Thailand for use in people aged 12–17; however, some unintentional misuse occurred.

Figure S1 Percentage of brands of COVID-19 vaccine distribution of booter doses by age groups in source population (N= 33,394,922)

Table S3 Characteristic of the study population (n= 2,450: case 490, control 1960)

Variables	*Categories*	*Case (n=490)*	*Control (n=1,960)*	*p-value**
Sex	Male	245 (50.0%)	888 (45.3%)	0.06
	Female	245 (50.0%)	1072 (54.7%)	-
Age. median. year (IQR)	Cont.	55 (40, 69)	44 (31, 57)	**<0.01**
Age group	12-20 years	17 (3.5%)	123 (6.3%)	**0.02**
	21-30 years	56 (11.4%)	361 (18.4%)	**<0.01**
	31-40 years	52 (10.6%)	377 (19.2%)	**<0.01**
	41-50 years	78 (15.9%)	404 (20.6%)	**0.02**
	51-60 years	93 (19.0%)	298 (15.2%)	**0.03**
	>60 years	190 (38.8%)	397 (20.3%)	**<0.01**
	Missing	4 (0.8%)	0 (0.0%)	-
Brands of booster vaccine	AstraZeneca┼	138 (28.2%)	343 (17.5%)	**<0.01**
	Moderna	41 (8.4%)	333 (17.0%)	**<0.01**
	Pfizer	311 (63.5%)	1284 (65.5%)	0.40
Underlying diseases	Metabolic syndromes	60 (12.2%)	171 (8.7%)	**0.02**
	Other diseases	13 (2.7%)	50 (2.6%)	0.90
	Unreported	417 (85.1%)	1739 (88.7%)	**0.03**
Occupation	Frontline workers	86 (17.6%)	109 (5.6%)	**<0.01**
	Non-frontline workers	404 (82.4%)	1851 (94.4%)	-
Regimens of booster dose	Heterologous doses	116 (23.7%)	534 (27.2%)	0.11
	Heterologous booster dose	294 (60.0%)	1064 (54.3%)	**0.02**
	Homologous booster dose	80 (16.3%)	362 (18.5%)	0.27
Period after booster dose policy initiation	<3 month	70 (14.3%)	27 (1.4%)	**<0.01**
	3-6 months	120 (24.5%)	405 (20.7%)	0.07
	6-9 months	237 (48.4%)	1184 (60.4%)	**<0.01**
	>9 months	63 (12.9%)	344 (17.6%)	**0.01**
Period after vaccine introduction	<3 month	56 (11.4%)	198 (10.1%)	0.39
	3-6 months	218 (44.5%)	799 (40.8%)	0.14
	6-9 months	126 (25.7%)	568 (29.0%)	0.15
	>9 months	90 (18.4%)	395 (20.2%)	0.38
Dose interval	<3 month	89 (18.2%)	194 (9.9%)	**<0.01**
	3-6 months	333 (68.0%)	1,489 (76.0%)	**<0.01**
	>6 months	68 (13.9%)	277 (14.1%)	0.89

**Pearson's chi-squared test for categdorical variables, Wilcoxon rank-sum test for continuous variables* *Data are n (%), unless otherwise indicated*

┼*AstraZeneca was not recommended in Thailand for use in people aged 12–17; however, some unintentional misuse occurred.*

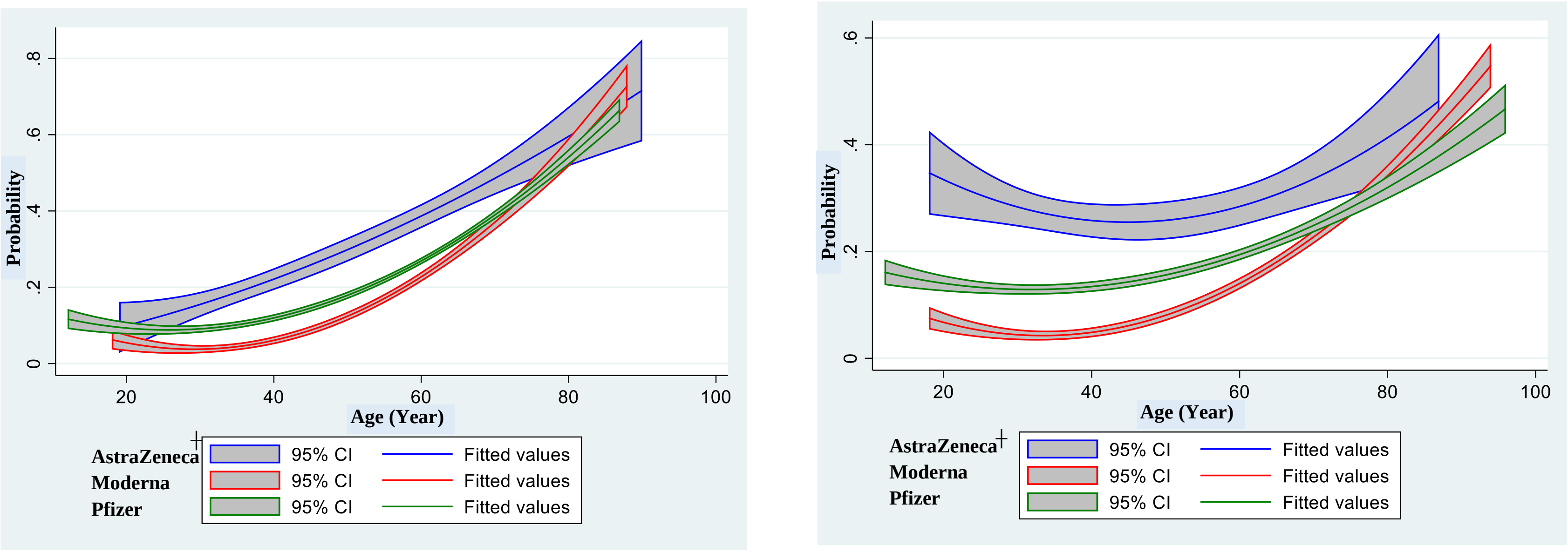

Male **Female**

+AstraZeneca was not recommended in Thailand for use in people aged 12–17; however, some unintentional misuse occurred.

Figure S2 Probability of reported SAE after a booster dose of COVID-19 vaccination across ages stratified by brands of booster dose among males (left) and females (right)
